# Characteristics and Absorption Rate of Whey Protein Hydrolysates Prepared Using Flavourzyme after Treatment with Alcalase and Protamex

**DOI:** 10.3390/molecules28247969

**Published:** 2023-12-06

**Authors:** Yeok Boo Chang, Hyeongyeong Kim, Se Kyung Lee, Hye-Jin Kim, A-Hyun Jeong, Hyung Joo Suh, Yejin Ahn

**Affiliations:** 1 Department of Integrated Biomedical and Life Science, Graduate School, Korea University, Seoul 02841, Republic of Korea; fjuae4@korea.ac.kr (Y.B.C.); hyunkyung999@korea.ac.kr (H.K.);; 2 Transdisciplinary Major in Learning Health Systems, Department of Healthcare Sciences, Graduate School, Korea University, Seoul 02841, Republic of Korea; 3 R&D Group, Maeil Health Nutrition Co., Ltd., Pyeongtaek 17714, Republic of Korea; hyejink@maeil.com (H.-J.K.); ah.jeong@maeil.com (A.-H.J.)

**Keywords:** absorption, Caco-2 cell, digestibility, hydrolysate, whey protein

## Abstract

The purpose of this study was to evaluate the physicochemical properties of whey protein hydrolysate and determine changes in absorption rate due to enzymatic hydrolysis. The molecular weight distribution analysis of whey protein concentrate (WPC) and low-molecule whey protein hydrolysate (LMWPH) using the Superdex G-75 column revealed that LMWPH is composed of peptides smaller than those in WPC. Fourier-transform infrared spectroscopy indicated differences in peak positions between WPC and LMWPH, suggesting hydrolysis-mediated changes in secondary structures. Moreover, LMWPH exhibited higher thermal stability and faster intestinal permeation than WPC. Additionally, oral LMWPH administration increased serum protein content at 20 min, whereas WPC gradually increased serum protein content after 40 min. Although the total amount of WPC and LMWPH absorption was similar, LMWPH absorption rate was higher. Collectively, LMWPH, a hydrolysate of WPC, has distinct physicochemical properties and enhanced absorptive characteristics. Taken together, LMWPH is composed of low-molecular-weight peptides with low antigenicity and has improved absorption compared to WPC. Therefore, LMWPH can be used as a protein source with high bioavailability in the development of functional materials.

## 1. Introduction

Whey, a by-product of cheese production, accounts for approximately 20% of milk protein and is considered a protein source [1]. Whey protein has a variety of nutritional and physiological functions. It contains four major proteins: α-lactalbumin (α-LA), β-lactoglobulin (β-LG), immunoglobulins, and bovine serum albumin (BSA) [2,3]. Whey hydrolysate reduces antigenicity through enzymatic hydrolysis and is considered a low-allergenic functional material [4]. Whey hydrolysate obtained by treating whey protein with proteolytic enzymes such as trypsin and papain contains bioactive peptides with various physiological functions. The active peptides present in whey protein hydrolysate are dependent on the enzyme used for hydrolysis [5]. Bioactive peptides include opioid peptides, angiotensin I-converting enzyme inhibitory peptides, antithrombotic peptides, immunomodulatory peptides, mineral transport peptides, antioxidant peptides, and peptides with muscle loss inhibitory activity [6,7].

Dipeptides and tripeptides contained in protein hydrolysates are absorbed faster than proteins or amino acids and effectively supply protein [8,9]. Previous studies have shown that low molecular whey protein hydrolysate (LMWPH), which is hydrolyzed from whey protein concentrate (WPC) using proteolytic enzymes, contains a large amount of branched amino acids. LMWPH has shown to promote muscle protein synthesis via the PI3K/Akt/mTOR pathway and inhibit muscle protein degradation via the PI3K/Akt/Foxo3a pathway in C57BL/6 mice [10]. In addition, determining the stability of bioactive peptides is crucial because they may interact with other food components or be further hydrolyzed during gastrointestinal digestion [11,12]. After absorption, peptides are hydrolyzed by peptidases in enterocytes and proteases in the bloodstream, which can affect their bioactivity [13]. Therefore, it is important to evaluate the bioavailability as well as the physiological activity of protein hydrolysates before and after hydrolysis.

The purpose of this study was to investigate the physicochemical properties and digestion and absorption rates of whey proteins after enzymatic hydrolysis. LMWPH was prepared through combined treatment of three proteases (Alcalase, Protamex, and Flavourzyme) in WPC. The molecular weight distribution of WPC and LMWPH was confirmed through size exclusion chromatography. In addition, changes in secondary structure due to hydrolysis were evaluated using Fourier-transform infrared spectroscopy (FT-IR) analysis, and thermal properties were evaluated using differential scanning calorimetry (DSC). Caco-2 cell and intestinal permeability of WPC and LWMPH were investigated according to digestive enzyme treatment, and serum absorption rate was evaluated through single administration in SD rats.

## 2. Results

### 2.1. Molecular Weight Distribution of WPC and LMWPH

**Figure 1:**
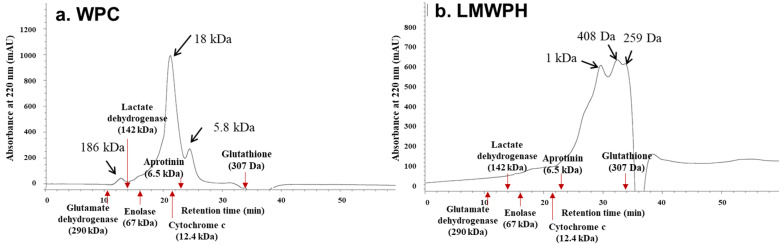
Molecular weight distribution of whey protein concentrate (WPC) (**a**) and low-molecule whey protein hydrolysate (LMWPH) (**b**). Absorbance at 220 nm was measured after eluting with 50 mM ammonium formate buffer (pH 5.5). Glutathione (MW 307), aprotinin (MW 6512), cytochrome c (MW 12,384), enolase (MW 67,000), lactate dehydrogenase (MW 142,000), and glutamate dehydrogenase (MW 290,000) were used as molecular weight standard proteins. WPC, whey protein concentrate; LMWPH, low-molecule whey protein hydrolysate.

The molecular weight distribution of WPC and LMWPH was analyzed using the Superdex G-75 column. Analysis of WPC revealed a large peak at 18 kDa and small peaks at 186 kDa and 5.8 kDa (Figure 1a), while that of LMWPH revealed large peaks at 1.0 kDa, 408 Da, and 259 Da (Figure 1b). LMWPH is composed of peptides smaller than those in WPC. The peak at 18 kDa in the WPC sample is attributed to β-LG, whose content is known to be the highest in whey protein; however, this peak was not detected in the LMWPH sample.

### 2.2. FT-IR Spectroscopy Analysis of WPC and LMWPH

**Figure 2:**
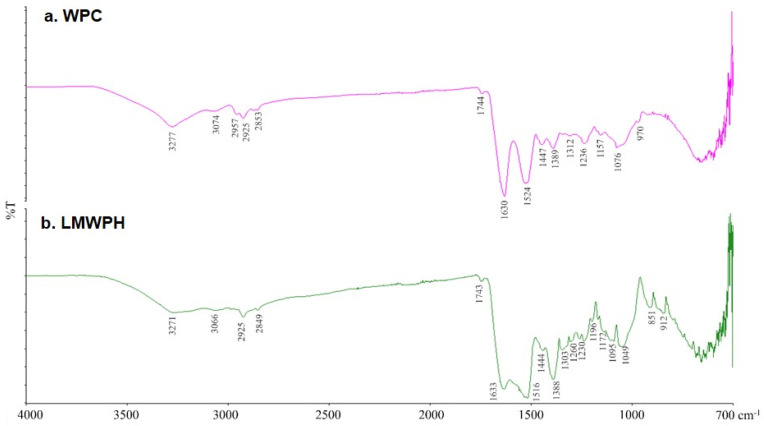
Fourier-transform infrared spectroscopy (FT−IR) spectra of (**a**) WPC and (**b**) LMWPH.

FT-IR analysis of WPC showed large peaks at 1630 and 1524 cm^−1^, whereas that of LMWPH showed peaks at 1633 and 1516 cm^−1^ (Figure 2). Both samples showed peaks in the 1600–1700 cm^−1^ region, which corresponds to amide I. Similarly, both samples showed peaks in the 1500–1600 cm^−1^ region, which corresponds to amide II. In addition, WPC showed peaks at 1157, 1312, 1389, and 1447 cm^−1^, while LMWPH showed peaks at 1172, 1196, 1230, 1303, and 1444 cm^−1^, all of which are attributed to amide III (1100–1500 cm^−1^). Collectively, WPC and LMWPH exhibited different peaks in amide I, II, and III regions. WPC and LMWPH display broad peaks at 3277 cm^−1^ and 3271 cm^−1^ with small shoulder peaks at 3074 cm^−1^ and 3066 cm^−1^, respectively. WPC and LMWPH also each showed a small peak at 2925 cm^−1^.

The content of secondary structures in WPC and LMWPH in the 1600–1700 cm^−1^ region (amide I region) was analyzed using the Prota−3S™ software (Table 1). The content of β-sheet in WPC was 36.41%, whereas that in LMWPH was decreased to 28.20%. Moreover, the α-helix structure content in LMWPH was 35.61%, which was higher than the content of other secondary structures. The hydrolysis-mediated changes in the secondary structure content are likely owing to the degradation of β-LG and α-LA, which are the main proteins in WPC.

### 2.3. Thermal Properties of WPC and LMWPH

The thermal properties of WPC and LMWPH were analyzed using DSC (Figure S1 and Table 2). The thermal stability of WPC was slightly lower than that of LMWPH, as indicated by the onset temperature (T_s_) and end temperature (T_e_) of peaks 1 and 2. The denaturation temperature (T_max_) at peak 1 was 53.23 °C for WPC and 94.99 °C for LMWPH, while T_max_ at peak 2 was 120.81 °C for WPC and 164.14 °C for LMWPH. These findings suggest that hydrolysis increased the thermal stability of LMWPH.

### 2.4. In Vitro Digestion

**Figure 3:**
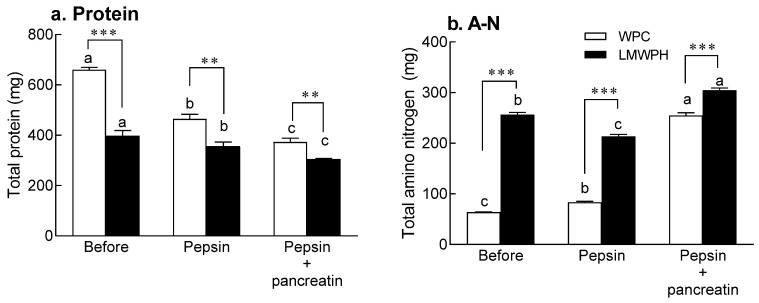
Total protein and amino nitrogen content in gastrointestinal-digested WPC and LMWPH in vitro. Data are expressed as the mean ± standard deviation (*n* = 3). Different letters (a–c) indicate statistical significance at the *p* < 0.05 level according to Tukey’s post hoc test. Additionally, the significant differences between the WPC group and the LMWPH group were as follows: ** *p* < 0.01 and *** *p* < 0.001 (Student’s *t*-test). WPC, whey protein concentrate; LMWPH, low-molecule whey protein hydrolysate.

The total protein content in WPC and LMWPH was measured before and after treatment with digestive enzymes (pepsin and pancreatin; Figure 3a). The total protein content in WPC and LMWPH before digestive enzyme treatment was significantly higher than that after digestive enzyme treatment (*p* < 0.05). Moreover, the protein content in WPC was significantly higher than that in LMWPH before digestive enzyme treatment (*p* < 0.001; Figure 3a). The digestive enzymes significantly decreased the protein content in a time-dependent manner (*p* < 0.05; Figure 3a).

In addition, digestive enzymes increased the amino nitrogen content in WPC by 299.3% from 63.82 to 254.84 mg. However, as LMWPH is already hydrolyzed by enzymes, the increase in amino nitrogen content by digestive enzymes was small. As WPC was composed of high-molecular weight peptides, the digestive enzyme treatment increased the amino nitrogen content. These results suggest that the active peptides contained in LMWPH were resistant to digestive enzymes.

### 2.5. Analysis of Intestinal Permeability of WPC and LMWPH before and after Digestive Enzyme Treatment Using Caco-2 Cells

**Figure 4:**
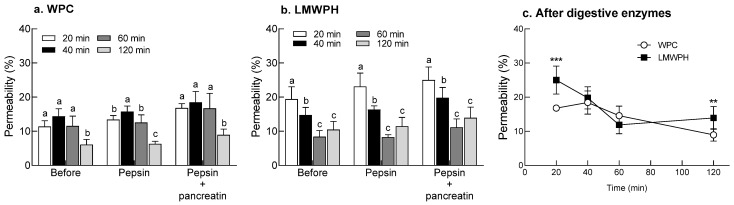
Caco-2 cell permeability of gastrointestinal-digested WPC and LMWPH in vitro. Data are expressed as the mean ± standard deviation (n = 4). Different letters (a–c) indicate statistical significance at the *p* < 0.05 level according to Tukey’s post hoc test, with no statistical significance between groups sharing the same letter. Additionally, the significant differences between the WPC group and the LMWPH group were as follows: ** *p* < 0.01 and *** *p* < 0.001 (Student’s *t*-test). WPC, whey protein concentrate; LMWPH, low-molecule whey protein hydrolysate.

Changes in Caco-2 cell permeability of WPC and LMWPH according to digestive enzyme treatment were evaluated (Figure 4). WPC showed a high Caco-2 cell permeability at 40 min in all digestive enzyme processes, and in particular, pepsin treatment showed a significantly higher cell permeability than other treatment times (*p* < 0.05, Figure 4a). At 40 min of WPC treatment, the Caco-2 cell permeability increased with the pepsin and pancreatin combination treatment (18.45%) compared to before the digestive enzyme treatment (14.36%). LMWPH showed significantly higher Caco-2 cell permeability at 20 min than other treatment times in all digestion processes (*p* < 0.05, Figure 4b). At 20 min of LMWPH treatment, the pepsin and pancreatin combination treatment (25.01%) showed higher intestinal permeability than before digestive enzyme administration (19.40%). After treatment with digestive enzymes (pepsin and pancreatin), the Caco-2 cell permeability of WPC and LMWPH was compared according to treatment time. LMWPH had significantly higher intestinal permeability than WPC at both 20 and 120 min of treatment (*p* < 0.001 and *p* < 0.01, respectively, Figure 4c). There was no difference in Caco-2 cell permeability between WPC and LMWPH at 40 and 60 min of treatment. As a result, LMWPH showed higher intestinal permeability in a faster time than WPC.

### 2.6. Intestinal Permeability of WPC and LMWPH after Digestive Enzyme Treatment Using the Intestinal Sac

**Figure 5:**
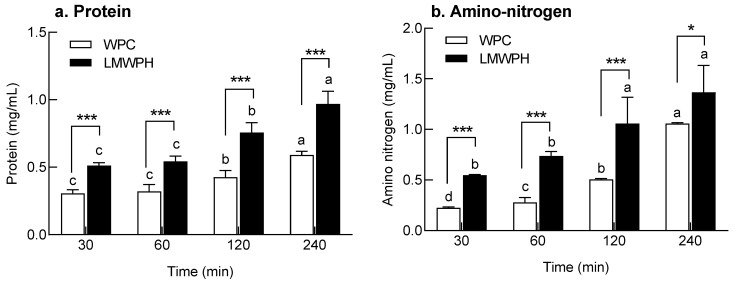
Changes in the content of permeated protein and amino nitrogen in the WPC and LMWPH groups using the intestinal sac model. Data are expressed as the mean ± standard deviation (n = 3). Different letters (a–c) indicate statistical significance at the *p* < 0.05 level according to Tukey’s post hoc test, with no statistical significance between groups sharing the same letter. Additionally, the significant differences between the WPC group and the LMWPH group were as follows: * *p* < 0.05 and *** *p* < 0.001 (Student’s *t*-test). WPC, whey protein concentrate; LMWPH, low-molecule whey protein hydrolysate.

Intestinal permeability of WPC and LMWPH after digestive enzyme treatment was assessed using the intestinal sac (Figure 5). The protein and amino nitrogen content permeating the intestinal sac increased with increasing intestinal transit time. Moreover, the protein and amino nitrogen permeation pattern according to the permeation time was similar for both WPC and LMWPH. Permeation of protein (*p* < 0.001; Figure 5a) and amino nitrogen (*p* < 0.001 and *p* < 0.05, respectively, Figure 5b) in LMWPH was significantly higher than the permeation of those in WPC at all-time points. Collectively, the intestinal sac model also confirmed that intestinal permeability of LMWPH was higher than that of WPC.

### 2.7. Evaluation of the Absorption Rate of the Samples after a Single High-Dose Administration to SD Rats

**Figure 6:**
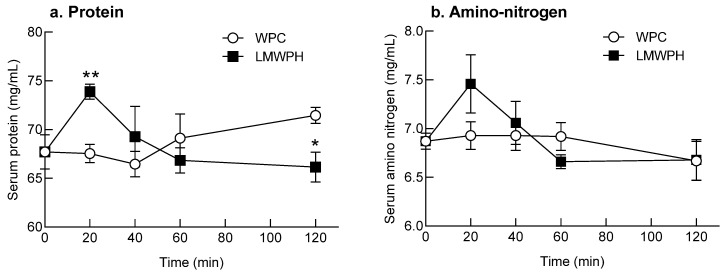
Changes in the content of serum protein and amino nitrogen after oral administration of LMWPH and WPC to Sprague Dawley rats. Data are expressed as the mean ± standard error of mean. The significant difference between the WPC and LMWPH groups is shown as * *p* < 0.05 and ** *p* < 0.01 (Student’s *t*-test). WPC, whey protein concentrate; LMWPH, low-molecule whey protein hydrolysate.

Serum protein and amino nitrogen content was measured at different time points after oral administration of WPC and LMWPH (Figure 6). Serum protein content was highest 20 min after oral administration of LMWPH and gradually decreased thereafter. In contrast, for WPC, serum protein content gradually increased after 40 min of oral administration (Figure 6). Furthermore, serum protein content was significantly higher 20 min after oral administration of LMWPH than WPC (*p* < 0.01; Figure 6a). On the contrary, serum protein content was significantly higher 120 min after oral administration of WPC than LMWPH (*p* < 0.05, Figure 6a).

The LMWPH group showed highest serum amino nitrogen content 20 min after oral administration and then a gradual decrease. On the contrary, the WPC group showed similar amino nitrogen content between 20 and 60 min of oral administration and then a decrease after 120 min (Figure 6b). After 20 min of oral administration, amino nitrogen content was considerably higher in the LMWPH group than in the WPC group (Figure 4b). The absorption rate 20 min after a single oral administration of LMWPH was high, whereas that of WPC was low.

The area under the curve (AUC) for protein absorption after oral administration of WPC and LMWPH was similar at 8266 and 8199 mg·min/mL, respectively. Similarly, the AUC for amino nitrogen absorption after oral WPC and LMWPH administration was similar at 822.8 and 825.9 mg·min/mL, respectively.

## 3. Discussion

Bioactive peptides derived from whey proteins are produced by hydrolyzing proteins through heat treatment, chemical and enzymatic hydrolysis, and fermentation [14]. Enzymatic hydrolysis is the preferred extraction method, mainly using proteases such as Trypsin, Chymotrypsin, Pepsin, and Alcalase. Whey-derived peptides have physiological activities such as antioxidant, antibacterial, antihypertensive, and anti-inflammatory, and peptides with a wide range of physiological properties are produced through a combination of various enzymes [15]. Although whey protein is considered a relatively safe protein, concerns about milk-derived allergies still remain. However, low-molecular-weight whey peptides are known to have markedly reduced antigenicity [16], so it is necessary to extract low-molecular-weight whey-derived peptides from which the major antigen, β-LG, has been removed through enzymatic hydrolysis. LMWPH prepared by additional hydrolysis with Flavourzyme after treatment with Alcalase and Protamex is a potential material in the functional food field because of its muscle loss inhibitory activity [10]. Therefore, in this study, the characteristics and digestion and absorption rates of LMWPH were evaluated for its application in the development of functional foods.

The properties of whey protein hydrolysates are attributed to β-LG and α-LA, the main proteins in whey protein. α-LA, which accounts for approximately 20% of whey protein, contains eight cysteine residues that form four disulfide bonds. The α-helix and β-sheet regions in α-LA have a strong calcium binding force [17]. Furthermore, β-LG accounts for 65% of whey protein and consists of 162 amino acid residues, including five cysteine residues that exist in the form of two disulfide bonds and one free hydrogen sulfide [18]. β-LG is mainly composed of a β-sheet structure that is simpler than the α-helical form of α-LA [19]. Structural changes expected to occur in protein structure due to protein hydrolysis can be observed through FT-IR spectrum analysis. The peak at 2925 cm^−1^ is observed in the WPI and LMWPH, and this spectral band (2925 cm^−1^) is associated with N-H stretching vibrations [20]. The broad peaks in the 3700–2800 cm^−1^ range in both samples are mainly related to O-H stretching, C-H stretching, and residual moisture [21]. The shoulder at 3074 cm^−1^ and 3066 cm^−1^ in the WPC and LMWPH corresponds to the C = C-H stretching vibration [22]. Absorption in the amide I region was considered most useful for FT-IR analysis of the secondary structure of proteins. α-helix (1646–1664 cm^−1^), β-sheet (1615–1637 and 1682–1700 cm^−1^), β-turn (1664–1681 cm^−1^), and random coil (1637–1645 cm^−1^) are observed in the amide 1 region [23].

β-LG is more accessible to digestive enzymes than α-LA and can, therefore, be hydrolyzed more easily [17,24]. In this study, hydrolysis of LMWPH changed the secondary structure of the main proteins, decreasing the content of β-sheet structure and increasing the ratio of α-helix structure (Table 1). When the N-terminus is increased by the hydrolysis of whey protein by the endoproteases Alcalase and Protamex, smaller peptides appear to be hydrolyzed by the exoprotease Flavourzyme [25]. Here, the chromatogram of WPC in Figure 1a shows a peak at 18 kDa, which corresponds to β-LG; this peak was not observed for the LMWPH sample. Protein secondary structure determines chemical reactivity, intermolecular interactions, and functional properties [23]. Hydrolysis not only caused structural changes but also caused changes in the thermal properties of WPC and LMWPH (Figure S1 and Table 2). Heat treatment further loosens the protein structure and weakens the internal hydrophobic bond [26]. Additionally, protein denaturation by heat treatment reduces solubility through aggregation [27]. The turbidity of whey proteins increases due to the aggregation of β-Ig due to heat treatment, but the WPH treated with enzymes did not show changes in turbidity and viscosity due to the enzyme inactivation process [28]. Similarly, Oldfield et al. [29] reported that denaturation of IgG was induced during the high-temperature preheating step during the spray drying process, but changes in α-LA and β-LG were minimal. Enzymatic hydrolysis exposes hydrophobic amino acids inside the protein, increasing the surface hydrophobicity of the hydrolysate [30], which can change thermal properties. In particular, enzyme-treated whey protein hydrolysate has increased thermal stability as the degree of hydrolysis increases and shows high solubility even at acidic pH [27].

The evaluation of the digestion and absorption rates of LMWPH with these characteristics indicates its potential for commercial use. Moreover, analyzing digestibility using artificial digestive enzyme treatment and permeability using Caco-2 cells is time and cost-effective and allows significant correlation with in vivo studies of hydrolysates [31]. Digestion of proteins increases the amount of free amino acids and/or peptides [32]. In this study, the amino nitrogen content in WPC increased slightly when treated with pepsin, but increased rapidly when treated with pancreatin (Figure 3). Furthermore, β-LG has a folding structure and is resistant to pepsin because hydrophobic amino acids at the pepsin cleavage site form strong hydrophobic bonds inside the β-sheet structure [33,34]. However, α-LA is readily hydrolyzed by pepsin [35]. Here, pancreatic juice hydrolyzed β-LG, which has 15 and three residues of lysine and arginine, respectively, at the trypsin cleavage site [36], and rapidly increased the amino nitrogen content. LMWPH, which had already been hydrolyzed by enzymes, showed slight changes in the amino nitrogen content after digestive enzyme treatment.

Peptides undergoing digestion are absorbed by the epithelial cells in the small intestine; therefore, permeability analysis using Caco-2 cells can help predict intestinal absorption [37]. In this study, the permeability of LMWPH, which had a high amino nitrogen content, was highest at 20 min, whereas that of WPC was high at 40 min (Figure 4). In the intestinal sac model, LMWPH showed higher intestinal permeability than WPC (Figure 5). The size and hydrophobicity of peptides affect their permeation into intestinal epithelial cells. The bioavailability decreases rapidly when the molecular weight exceeds 500 Da [38]. LMWPH is composed of peptides smaller than those in WPC, and hydrolysis exposes the internal hydrophobic peptides to the surface; this may explain the difference in the permeation using the Caco-2 cell model. Changes in serum protein and amino-nitrogen after a single dose in the SD rat model showed that the absorption of LMWPH was high at 20 min after oral administration, but the absorption of WPC was slow (Figure 6). In the time-dependent absorption rate graph, the AUCs of protein and amino nitrogen for the WPC and LMWPH groups were similar, that is 8266 and 8199 mg·min/mL and 822.8 and 825.9 mg·min/mL, respectively. There was no significant difference in the AUC between WPC and LMWPH; however, the absorption rate of LMWPH was faster than that of WPC. Similarly, Farup et al. [39] reported that, depending on the degree of hydrolysis, whey protein markedly increases total plasma amino acids, and whey protein hydrolysate increases plasma total amino acids faster than casein. This rapid protein absorption contributes to a rapid rise in blood amino acid concentrations and acceleration of whole-body protein synthesis [40]. Slowly digested proteins are mostly absorbed from the intestines instead of increasing the concentration of blood amino acids absorbed into peripheral blood vessels. This results in reduced muscle protein synthesis compared to rapidly digested proteins [41]. Therefore, rapidly absorbed LMWPH stimulates muscle protein synthesis more effectively than slowly absorbed proteins. Indeed, oral administration of LMWPH to C57BL/6 mice for two weeks improved the total protein content by inhibiting muscle atrophy and promoting muscle differentiation caused by immobilization [10]. These results suggest that the active peptide contained in LMWPH reached the target cells following digestion and absorption and was involved in promoting muscle differentiation and inhibiting muscle loss. This study demonstrated that LMWPH composed of small-molecular weight peptides is absorbed faster than WPC and may contribute to the suppression of muscle loss caused by muscle atrophy.

In this study, LMWPH was prepared through enzymatic hydrolysis using a mixture of three proteases (Alcalse, Protamex, and Flavourzyme). Enzymatic treatment changed the secondary structure, degraded milk-derived allergenic β-LG, and improved the thermal stability of WPC. LMWPH showed higher Caco-2 cell and intestinal permeability than WPC, and a single high-dose administration of LMWPH to SD rats rapidly increased serum protein concentration. In conclusion, LMWPH has high bioavailability and can be used as an effective protein source in the development of functional materials and pharmaceuticals.

## 4. Materials and Methods

### 4.1. Sample Preparation

WPC and LMWPH used in the experiment were provided by Maeil Health Nutrition Co., Ltd. (Seoul, Republic of Korea). LMWPH was prepared as follows: WPC was dissolved in distilled water (*w*/*v* = 1:5), and the pH was adjusted to 7–7.5 using sodium bicarbonate. According to previous studies [42], Alcalase 2.4 L FG and Protamex were added at 0.2% of each substrate and incubated for 4 h, then Flavourzyme 1000 L was added at 0.2% of each substrate and incubated for 15 h. The temperature of the enzyme reaction was maintained at 50–55 °C, and all enzymes were purchased from Novozyme (Frederiksberg, Denmark). After the reaction was completed, the enzyme was inactivated (90 °C, 15 min), and the enzyme reaction solution was filtered and spray-dried to prepare whey protein hydrolysate (LMWPH).

### 4.2. Molecular Weight Distribution Analysis of WPC and LMWPH Using High-Performance Liquid Chromatography (HPLC)

The molecular weight distribution of WPC and LMWPH was analyzed using HPLC. WPC and LMWPH were filtered (Polyvinylidene fluoride, 0.45 μm) and 20 μL of the samples was injected into the Superdex G-75 column (10 × 300 mm, GE Healthcare, Anaheim, CA, USA). The mobile phase was 50 mM ammonium formate buffer (pH 5.5) at a flow rate of 0.5 mL/min, and absorbance was measured at 220 nm. The molecular weight distribution of the samples was determined according to the molecular weight distribution curve prepared using glutathione (MW 307), aprotinin (MW 6512), cytochrome c (MW 12 384), enolase (MW 67,000), lactate dehydrogenase (MW 142,000), and glutamate dehydrogenase (MW 29,000).

### 4.3. DSC Analysis of WPC and LMWPH

Thermal properties of WPC and LMWPH were analyzed using a PerkinElmer DSC 4 differential scanning calorimeter (PerkinElmer, Waltham, MA, USA) [43]. Samples (5 mg) were placed in an aluminum pan, sealed by compression, and then heated from 20 °C to 200 °C at a heating rate of 10 °C/min. The denaturation temperature and enthalpy value (ΔH) of the samples were analyzed using the Pyris 6.5 software (PerkinElmer).

### 4.4. FT-IR Spectroscopy Analysis of WPC and LMWPH

FT-IR spectra were obtained using the PerkinElmer Spectrum 100 FT-IR spectrometer (PerkinElmer) equipped with an attenuated total reflection (ATR) sampling accessory. Interferograms of eight scans were recorded from 4000 to 700 cm^−1^ at a resolution of 4 cm^−1^ using the ATR technique. Secondary structure analysis was performed as described by Carbonaro et al. [44] at 1700–1600 cm^−1^ (amide I region) using the Prota−3S™ software (BioTools, Jupiter, FL, USA). Spectral data were fitted to a Gaussian fixed band model using the second-order differentiation method to identify component peaks. After the r2 and F values of the fitted model were stabilized by performing iterative curve fitting, peaks were assigned to the corresponding features of the protein secondary structure and analyzed.

### 4.5. In Vitro Digestion

The digestion rate of WPC and LMWPH using artificial digestive enzymes was analyzed as previously described [43]. After dissolving 1 g of WPC or LMWPH in 10 mL of distilled water, the pH was adjusted to 2 using 5 N HCl. Then, pepsin (enzyme to substrate ratio of 1:35, *w*/*w*; Sigma-Aldrich, St. Louis, MO, USA) was added to the mixture and incubated at 37 °C for 1 h in a shaking incubator. The pH was adjusted to 7.5 using saturated NaHCO_3_ solution. Then, pancreatin (Sigma-Aldrich) was added to the mixture and incubated at 37 °C for 2 h. The reaction was terminated by heating the mixture at 100 °C for 15 min. The amino nitrogen and protein content was measured during the digestive enzyme treatment.

### 4.6. Intestinal Permeability Assay Using Caco-2 Cell Line

CacoReady 24-well plates (#KRECE-CCR04, Komabiotech, Seoul, Republic of Korea) were used for the intestinal permeability assay. The CacoReady plate contains a monolayer of Caco-2 cells that have been differentiated for 21 days on an insert in a transwell. CacoReady plates were thawed by incubating in a 5% CO_2_ incubator (37 °C) for 4 h. The medium was replaced with Dulbecco’s Modified Eagle’s Medium (DMEM) containing 1 g/L glucose, 10% fetal bovine serum, 1% glutamine, and 1% penicillin-streptomycin. Permeability experiments were performed on the fifth day after the plates were received and incubated with the medium, which was changed every two days. WPC and LMWPH before and after digestive enzyme treatment were diluted 10-fold with Hanks’ Balanced Salt Solution (HBSS) buffer and used for intestinal permeability analysis. After washing the Caco-2 plates thrice with HBSS buffer, samples (250 μL) were dispensed into the insert and 750 μL of HBSS buffer was dispensed into the receiver plate. The buffer in the receiver plate was assessed at 20, 40, 60, and 120 min to determine the total protein content. Permeability was analyzed as follows: permeability (%) = [total protein (mg) in the receiver plate]/[initial protein (mg) added to the insert] × 100.

### 4.7. Analysis of Protein and Amino Nitrogen Content

The protein content was analyzed using the bicinchoninic acid (BCA) method [45]. Briefly, 200 μL of BCA reagent was added to 25 μL of the sample and incubated at 37 °C for 30 min. Absorbance at 562 nm was measured. The protein content in the samples was calculated using a calibration curve, with BSA as the standard.

The amino nitrogen content was analyzed using the 2,4,6-trinitrobenzene sulfonic acid (TNBS) method [46]. Briefly, 400 μL of 0.212 M phosphate buffer (pH 8.2) and 400 μL of 0.1% TNBS were added to 50 μL of the sample and incubated at 50 °C for 1 h. Then, 800 μL of 0.1 M HCl was added followed by 1.6 mL of distilled water, and the absorbance at 340 nm was measured. The amino-nitrogen content was calculated, with L-leucine as the standard.

### 4.8. Intestinal Permeability Analysis Using the Intestinal Sac

The intestinal permeability of WPC and LMWPH was determined using the intestinal sac [47]. SD rats (male, 8 weeks old) were euthanized by CO_2_ inhalation, and 10 cm of the intestinal area near the cecum was removed and used as the intestinal sac. The intestinal sacs were filled with Krebs-Henseleit bicarbonate buffer and maintained at 37 °C with a gas mixture of 95% O_2_ and 5% CO_2_. Pepsin and pancreatin-treated WPC and LMWPH at a concentration of 10 mg/mL were injected (1 mL) into the intestinal sacs. Then, samples (10 mL) were collected at 30, 60, 120, and 240 min to measure the protein and amino nitrogen content in the permeate using the BCA and TNBS methods, respectively.

### 4.9. Evaluation of the Absorption Rate after a Single High-Dose Administration of Samples to SD Rats

An 8-week-old male SD rat (Oriental Bio, Seongnam, Republic of Korea) was allowed to adapt for one week prior to the experiment. Blood was collected 20, 40, 60, and 120 min after oral administration of 500 mg/kg WPC and LMWPH. Blood was collected alternately from the left and right jugular veins. Blood was centrifuged (2800× *g*, 15 min, 4 °C) to collect serum, and subsequently analyze total protein and amino nitrogen content.

## Figures and Tables

**Table 1:** Content of secondary structures in whey protein concentrate (WPC) and low-molecule whey protein hydrolysate (LMWPH).

Sample	α-Helix	β-Sheet	β-Turn	Random Coil
WPC	23.83 ± 7.60	36.41 ± 7.86	26.58 ± 8.08	13.18 ± 7.98
LMWPH	35.61 ± 10.74	28.20 ± 9.79	24.90 ± 10.37	11.29 ± 10.88

Data are expressed as the mean ± standard deviation. WPC, whey protein concentrate; LMWPH, low-molecule whey protein hydrolysate.

**Table 2:** Denaturation temperature and the associated enthalpy change (ΔH) of WPC and LMWPH.

Sample	Peak 1				Peak 2			
	T_s1_ (°C)	T_max1_ (°C)	T_e1_ (°C)	∆H_1_ (J/g)	T_s2_ (°C)	T_max2_ (°C)	T_e2_ (°C)	∆H_2_ (J/g)
WPC	41.73 ± 1.24	53.23 ± 1.32	65.49 ± 2.04	1.555 ± 0.07	76.18 ± 2.04	120.81 ± 3.07	164.07 ± 2.74	129.3 ± 5.64
LMWPH	56.96 ± 0.66	94.99 ± 2.48	127.8 ± 2.17	114.1 ± 5.91	143.49 ± 3.97	164.14 ± 4.11	178.85 ± 3.21	7.375 ± 0.04

Data are expressed as the mean ± standard deviation. WPC, whey protein concentrate; LMWPH, low-molecule whey protein hydrolysate; T_s1_, onset temperature; T_max_, maximum peak temperature; T_e_, end temperature.

## Data Availability

The data presented in this study are available within the article and Supplementary Materials. WPC and LMWPH samples are available from Maeil Health Nutrition Co., Ltd.

## Supplementary Materials

The following supporting information can be downloaded at: https://www.mdpi.com/article/10.3390/molecules28247969/s1, Figure S1: Differential scanning calorimetry thermograms of (a) whey protein concentrate (WPC) and (b) low-molecule whey protein hydrolysate (LMWPH).
