# Supplementary material for: Characteristics and Absorption Rate of Whey Protein Hydrolysates Prepared Using Flavourzyme after Treatment with Alcalase and Protamex
Source: Molecules. 2023 Dec 6;28(24):7969. doi: 10.3390/molecules28247969 (PMC10745520; doi:10.3390/molecules28247969)
Supplement: Supplementary file 1 [file molecules-28-07969-s001.zip › molecules-2695435-supplementary.pdf]

Supplementary data

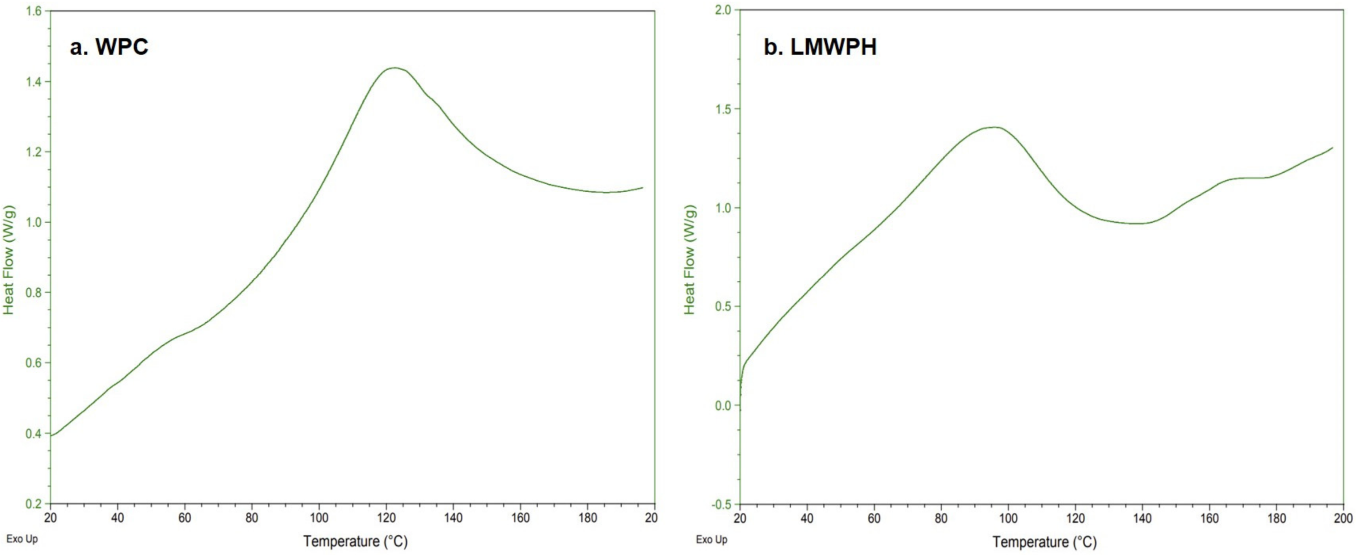

Figure S1. Differential scanning calorimetry thermogram of (**a**) whey protein concentrate (WPC) and (**b**) low-molecule whey protein hydrolysate (LMWPH). During scanning, the temperature was increased from 20 °C to 200 °C at a rate of 10 °C/min. WPC, whey protein concentrate; LMWPH, low-molecule whey protein hydrolysate.
